# Complete Genome Sequence Analysis of Acute and Mild Strains of Classical Swine Fever Virus Subgenotype 3.2

**DOI:** 10.1128/genomeA.01329-15

**Published:** 2016-01-28

**Authors:** Seong-In Lim, Song-Hee Han, HyeSook Hyun, Ji-Ae Lim, Jae-Young Song, In-Soo Cho, Dong-Jun An

**Affiliations:** Animal and Plant Quarantine Agency, Anyang, Gyeonggi-do, Republic of Korea

## Abstract

We report the complete genome sequences of two classical swine fever virus strains (JJ9811 and YI9908). Both belong to subgenotype 3.2. Strain JJ9811 causes mild symptoms and strain YI9908 causes acute symptoms. The sequences were 95.7% homologous at the nucleotide level and 95.6% homologous at the amino acid level.

## GENOME ANNOUNCEMENT

Classical swine fever virus (CSFV) belongs to the genus *Pestivirus* within the family *Flaviviridae*, which also includes border disease virus and bovine viral diarrhea viruses 1 and 2 (1). Acute classical swine fever (CSF) occurs mainly in young pigs and is characterized by high fever, lack of appetite, conjunctivitis, and constipation; these symptoms are often followed by diarrhea, neurological signs, and hemorrhage of the skin and other organs, possibly accompanied by severe thrombocytopenia and leucopenia (2). Host-virus interactions may involve many factors (host age, genetic background, immune status, herd sanitary status, and strain virulence), which can lead to different clinical outcomes (3). Since 2002, there has been a genotype shift from CSFV genotype 3 to CSFV genotype 2 in domestic pigs in South Korea (4). Strain JJ9811 isolated from Jeju Island in 1998 caused mild symptoms in pigs, whereas strain YI9908 isolated from the Yongin region was acutely virulent. Information about the complete genome of genotype 3 of CSFV is lacking. Therefore, the aim of this study is to analyze and compare the genome sequences of strains JJ9811 and YI9808 to improve our understanding of CSFV.

Total RNA was extracted from the blood of wild boar using the micro-column technique-based QIAamp Viral RNA minikit (QIAGEN, USA) and cDNA amplified with a one-step reverse transcription-PCR (RT-PCR) kit (QIAGEN, USA) using primers specific for CSFV genomic sequences (5, 6). The amplification products were then cloned into the pGEM-T plasmid and sequenced using T7 and SP6 primers and an ABI Prism 3730XI DNA sequencer.

The genome sequences of YI9908 and JJ9811 were 95.7% homologous at the nucleotide (nt) level and 95.6% homologous at the amino acid level. Comparative analysis of particular regions revealed rather low nt sequence homology: 90.1% for the Npro genes, 87.5% for the C genes, 96.6% for the Erns genes, 97.8% for the E1 genes, 93.2% for the E2 genes, 95.7% for the p7 genes, 96.3% for the NS3 genes, 94.8% for the NS4A genes, 95.8% for the NS4B genes, 95.7% for the NS5A genes, and 96.3% for the NS5B genes.

A similar analysis of 75 complete CSFV genome sequences deposited in GenBank revealed that the JJ9811 and YI9908 strains showed 88.9% and 89.5% nt sequence homology, respectively, with strain Alfort/187 (accession number X87939), which belongs to genotype 1. Also, the JJ9811 and YI9908 strains showed 84.2% and 83.9% nt sequence homology with strain YC11WB (accession number KC149990), which belongs to genotype 2.

A phylogenetic tree constructed using the Mega 6.01 program (7) and based on E2 partial (190 nt) sequences derived from 120 CSFV strains deposited in GenBank revealed that strains JJ9811 and YI9908 belong to genotype 3.2.

In summary, although strains JJ9811 and YI9908 belong to genotype 3.2, they show different levels of virulence. The information presented herein will be useful for future studies aimed at CSF eradication.

### Nucleotide sequence accession numbers.

The complete genome sequences of the JJ9811 and YI9908 strains have been deposited in GenBank under accession numbers KF669877 and KT716271, respectively.

## References

[1] Thiel HJ, Collett MS, Gould EA, Heinz FX, Houghton M, Meyers G, Purcell RH, Rice CM 2005 Family *Flaviviridae*, p 979–996. *In* Fauquet CM, Mayo MA, Maniloff J, Desselberger U, Ball LA (ed), Virus taxonomy. VIIIth report of the ICTV. Elsevier/Academic Press, London, United Kingdom.

[2] Bautista MJ, Ruiz-Villamor E, Salguero FJ, Sanchez-Cordon PJ, Carrasco L, Gomez-Villamandos JC 2002 Early platelet aggregation as a cause of thrombocytopenia in classical swine fever. Vet Pathol 39:84–91. doi:10.1354/vp.39-1-84. 12102222

[3] Floegel-Niesmann G, Bunzenthal C, Fischer S, Moennig V 2003 Virulence of recent and former classical swine fever virus isolates evaluated by their clinical and pathological signs. J Vet Med B 50:214–220. doi:10.1046/j.1439-0450.2003.00663.x. 12864895

[4] Song J-Y, Lim SI, Jeoung HY, Choi E-J, Hyun B-H, Kim B, Kim J, Shin Y-K, Dela Pena RC, Kim JB, Joo H, An DJ 2013 Prevalence of classical swine fever virus in domestic pigs in South Korea: 1999–2011. Transbound Emerg Dis 60:546–551. doi:10.1111/j.1865-1682.2012.01371.x. 22925439

[5] Park G, Lim S, Hong S, Song J 2012 Establishment and characterization of an infectious cDNA clone of a classical swine fever virus LOM strain. J Vet Sci 13:81–91. doi:10.4142/jvs.2012.13.1.81. 22437540 PMC3317462

[6] Leifer I, Hoffmann B, Höper D, Bruun Rasmussen T, Blome S, Strebelow G, Höreth-Böntgen D, Staubach C, Beer M 2010 Molecular epidemiology of current classical swine fever virus isolates of wild boar in Germany. J Gen Virol 91:2687–2697. doi:10.1099/vir.0.023200-0. 20660149

[7] Tamura K, Stecher G, Peterson D, Filipski A, Kumar S 2013 MEGA6: molecular evolutionary genetics analysis version 6.0. Mol Biol Evol 30:2725–2729. doi:10.1093/molbev/mst197. 24132122 PMC3840312

